# Protecting the Nerve Coaptation: Connector-Assisted Nerve Repair in Complex Injuries

**DOI:** 10.1016/j.jhsg.2025.100816

**Published:** 2025-08-23

**Authors:** Nesreen Zoghoul Alsmadi, Curt Deister, Peter Evans, Tamer Ghanem, Brandon Smetana, Deana Mercer

**Affiliations:** ∗ Axogen Corporation, Research & Development, Tampa, FL; † Cleveland Clinic Martin Health, Orthopaedic Surgery, Rehabilitation, and Sports Therapy, Stuart, FL; ‡ Premier Head and Neck Surgery, Flint, MI; § Indiana Hand to Shoulder Center, Indianapolis, IN; ‖ The University of New Mexico, UNM Orthopedics & Rehabilitation, Albuquerque, NM

**Keywords:** Conduit-assisted repair, Connector-assisted repair, Nerve injury, Nerve tube, Peripheral nerve repair

## Abstract

**Purpose:**

This study evaluated differences in outcomes of peripheral nerve repair using connector-assisted repair (CAR) or direct repair (DR) in an injured soft tissue bed.

**Methods:**

The sciatic nerve of the right leg in 20 male Lewis rats was exposed and transected. We simulated a traumatized wound bed by cauterizing the underlying muscle bed with a bipolar coagulator. Nerves were repaired with either DR or CAR using porcine small intestine submucosa conduits. At 6 weeks, adhesions were assessed semiquantitiatively, and the gastrocnemius wet muscle weight of each hind limb was recorded to evaluate muscle atrophy. Histology of the nerve was evaluated immediately distal to the nerve repair site. Data were analyzed for differences between repair methods.

**Results:**

The DR group had a considerably higher area of foamy phagocytes and CD68-stained macrophages than that of the CAR group. There were considerably more blood vessels and axons in the CAR group than in the DR group. There were no differences between DR and CAR with respect to gastrocnemius muscle wet weight, extraneural adhesions, or intraneural collagen-to-cell ratio.

**Conclusions:**

There was less area occupied by macrophages and foamy phagocytes in the CAR group, which was indicative of lower inflammatory response and resolving Wallerian degeneration. The CAR group also had more blood vessels and axons compared to that of the DR group, indicating more robust nerve regeneration. Gastrocnemius muscle weight between groups was similar, indicating that nerve regeneration was incomplete in both groups at the 6-week timepoint. These results highlight the potential benefits of CAR in protecting the nerve during the healing process.

**Clinical relevance:**

This in vivo study evaluates histological changes in peripheral nerves during regeneration following transection with either CAR or DR.

Achieving clinically meaningful recovery after repair of a lacerated peripheral nerve is dependent upon the extent of the injury, repair technique, surgeon skill, and unimpeded regeneration. Successful axon regeneration requires timely resolution of Wallerian degeneration, minimal intraneural fibrosis at the neurorrhaphy site, and minimal extraneural adhesion.^1^^,^^2^ Extraneural adhesions are detrimental to recovery because nerve tethering hinders gliding and/or creates constriction when nerves are stretched during limb motion.^1^^,^^2^ These adhesions can have secondary detrimental effects that impact the patient’s quality of life including neuropathic pain, reduced mobility, and impaired nerve regeneration.^3^^,^^4^ Extraneural adhesions can hinder nerve gliding, reduce mobility, and compress nerves, causing ischemia and mechanical damage to nerve fibers and their protective myelin sheath, which, over time, leads to intraneural fibrosis—scar tissue formation within the nerve itself. This intraneural scar forms a physical barrier to nerve regeneration that disrupts nerve signal transmission, contributes to neuropathic pain, causes sensory disturbances, and entraps regenerating nerve fibers, leading to symptomatic neuroma formation.^3^^,^^5^ This intricate interplay of maintaining gliding of the nerve, particularly when adjacent to joints, and facilitating effective nerve regeneration underlines the complex challenges faced in treating nerve injuries and the critical importance of strategies aimed at minimizing scar formation within and around the nerve.

Animal studies that evaluate the efficacy of surgical nerve repair techniques provide meaningful empirical nerve regeneration data. In one such animal study, Zoghoul Alsmadi et al^6^ found that inducing an injury of the soft tissue surrounding the nerve with a bipolar coagulator resulted in an elevated inflammatory response, intraneural scar, and extraneural adhesions. We hypothesize that these responses may be circumvented if a nerve is surrounded with an anti-adhesion barrier after injury to the nerve and soft tissue bed. Such anti-adhesion barriers around the nerve include autologous fat flaps; off-the-shelf allogenic, xenogenic, or synthetic conduit; or a combination of these options.^3^^,^^7^ These physical barriers prevent scar tissue from adhering to the nerve and provide an environment that supports nerve regeneration.^8^

We specifically investigated the use of a nerve conduit as a barrier to scar and inflammation after nerve and soft tissue trauma. Connector-assisted repair (CAR; ie, using a nerve conduit at the site of a nerve coaptation) has been shown to provide mechanical support, reduce extraneural and intraneural fibrosis, reduce tension at the nerve coaptation site, and provide a favorable microenvironment that facilitates axonal growth and remyelination.^9^, ^10^, ^11^, ^12^ The nerve coaptation is the focal site of nerve healing, which makes it susceptible to scarring, potentially even more so when performed adjacent to damaged tissues. We hypothesize that protecting the coaptation using a conduit at the site of repair results in different outcomes when compared to direct repair (DR) alone.

## Materials and Methods

Twenty male Lewis rats aged 8–10 weeks and weighing 200–300 g were used for the study. All surgical procedures and animal care conformed to National Institutes of Health guidelines, were performed in accordance with the standard of care practices at the University of South Florida Morsani College of Medicine and Heart Institute, and approved by the University of South Florida Institutional Animal Care and Use Committee. Animals were randomly assigned to either the DR group (n = 10) or the CAR group (n = 10). Sample size was determined based on power analysis using pilot data evaluating CD68 percent area and foamy cells percent area, with power analysis (α = 0.05, power = 0.90) requiring a minimum of four animals per group.

### Surgical procedures

**Figure 1:**
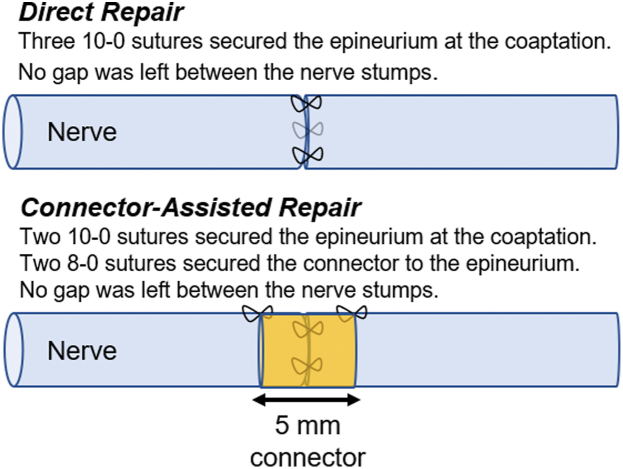
Surgical technique of direct repair (DR) and connector-assisted repair (CAR) groups.

All surgical procedures were performed by a single scientist (C.D.) highly trained in microsurgery using a surgical microscope (Leica model F12, Leica Microsystems Inc), which was used to enhance the magnification of the surgical field. Prior to surgeries, all animals received buprenorphine at 1 mg/kg and meloxicam at 2 mg/kg as a premedication and postsurgery for the first 3 days. During surgery, animals were kept under anesthesia using isoflurane, initially set at 3% and gradually reduced to 1%. In all rats, the sciatic nerve was exposed and circumferentially released from the surrounding soft tissues at the midthigh level using surgical neurolysis. The soft tissue surrounding the nerve was cauterized with a bipolar coagulator, as described previously, to induce tissue disruption and create a scarred wound bed.^6^ The tips of the bipolar coagulator forceps were fixed at 5 mm apart using a 3-dimensional printed holder, which was held at a 45° angle to the soft tissue. The tissue was cauterized with the bipolar coagulator for 3 seconds at 35 watts. Then, the forceps were advanced along the edge of the tissue with consistent spacing between cauterized areas until a muscle injury measuring 10 × 20 mm was created. The area of cauterization was 7 mm distal to the iliac fascia and positioned so that the sciatic nerve would be centered within the 10 × 20 mm disrupted wound bed. The nerve was transected with a 15 blade, 12 mm from the iliac fascia, positioning the transected region in the center of the zone of injury. In the DR group, the nerves were repaired by suturing the epineurium of the proximal and distal stumps together with three simple interrupted 10-0 nylon nonresorbable sutures, evenly placed around the nerve, approximately 120° apart. In the CAR group, the nerves were repaired by suturing the epineurium of the proximal and distal stumps together with two simple interrupted 10-0 nylon nonresorbable sutures, evenly placed 180° apart. A 1.5 × 5 mm nerve connector (Axoguard Nerve Connector, Axogen Corporation) was placed over the repair site, ensuring that the coaptation was centered within the nerve connector. The nerve connector was sutured to the epineurium on each end of the nerve using one simple interrupted 8-0 nonresorbable nylon suture (Fig. 1). Each surgical site was closed using standard surgical technique. Animals recovered for 6 weeks.

### End point procedures

At 6 weeks after surgery, rats were humanely killed using medical grade carbon dioxide at 100% CO_2_ gas at 30% to 70% of the chamber volume per minute to reduce distress. Euthanasia was evaluated by involuntary initiation of micturition and bulging/loss of eye color as well as observation of cessation of cardiovascular and respiratory movements. Euthanasia was confirmed by thoracotomy to evaluate cessation of heartbeat. Adhesion assessments were performed by the same scientist who performed the repair using a previously defined assessment scale (originally described by Kokkalis et al^8^) and an assessment of nerve mobility (Table).^6^ Gastrocnemius muscles were collected from both legs in each animal, and the wet weight of injured and contralateral gastrocnemius muscles was recorded.

**Table 1:** Nerve Adhesion Scoring, Performed Prior to Sample Explant

Category	Evaluation Criteria and Description		Score
Nerve immobility, tested by pulling on the nerve	Nerve moves as freely as healthy nerve in the contralateral leg		0
	Nerve movement is restricted by scar tissue, pulling surrounding tissues		1
	Nerve is completely immobilized by the scar tissue		2
Adhesion quality (Circle all that apply, select the highest score, do not add scores, highest score is selected and recorded, max = 4)	None: similar to healthy contralateral nerve		0
	Appearance	Transparent	1
		Translucent	2
		Opaque	3
	Vascularization	Avascular	1
		Capillaries present	3
		Large vessels present	4
Tenacity (max = 3)	None: similar to healthy contralateral nerve		0
	Adhesions fall apart with blunt dissection (applying gentle traction using forceps)		1
	Adhesions tear with considerable traction (using forceps to pull two ends but does not need cutting)		2
	Adhesions require sharp transection (require transection using scissors)		3
Extent of site involvement for adhesions	None: similar to healthy contralateral nerve		0
	<50% of the exposed nerve		2
	>50% of the exposed nerve		4
Total Adhesion Score: cumulative of nerve immobility, adhesion quality, tenacity, and extent of site involvement for adhesions	Add the highest score from each assessment criteria above (nerve immobility, adhesion quality, tenacity, and extent of site involvement for adhesions)		0–13

### Histology

**Figure 2:**
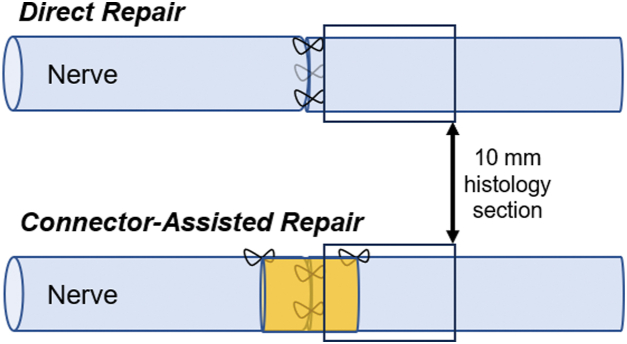
Schematic of distal nerve stump explants for DR and CAR groups.

Control samples of uninjured contralateral nerve were included for benchmarking “healthy nerve.” Samples of the repaired injured sciatic nerve and the surrounding soft tissue were recovered and included a nerve segment from 10 mm proximal to the injury site to 10 mm distal to the injury site. All samples were marked with a tissue marker or suture on the proximal nerve stump. The tissue was fixed in 10% neutral buffered formalin for a minimum of 48 hours. Samples were embedded in paraffin blocks. The distal nerve stump sections were collected approximately 1.5 mm distal to the coaptation site (Fig. 2).

The distal nerve stump was sectioned transversely in 5 μm sections. Nerve stump sections were stained with Masson’s trichrome (MT) to evaluate collagen-to-cell ratio and foamy phagocytes, anti-CD68 to evaluate macrophage cell response, anti-α smooth muscle actin (αSMA) to evaluate vascularization, as well as anti-neurofilament (NF) and anti-myelin basic protein (MBP) to evaluate axons. Immunohistochemistry slides were prepared after sectioning as follows: Heat mediated antigen-retrieval was performed by placing the slides in sodium citrate buffer (pH 6; Abcam). Peroxidase activity and nonspecific protein binding were quenched using Bloxall (Abcam) and 10% normal goat serum with 1% bovine serum albumin in tris buffered saline, respectively. Slides were then stained with antibodies to one or more of the following: CD68 (ab125212, 1:500 dilution, Abcam), αSMA (ab124964, 1:1000, Abcam), MBP (ab40390, 1:100 dilution, Abcam), and NF (N2787, 1:200 dilution, Sigma).

For CD68 staining, incubation with goat anti-rabbit immunoglobulin G heavy and light chains horseradish peroxidase secondary antibody (ab205718, 1:1000 dilution, Abcam) was followed by exposure to diaminobenzidine (DAB, BDB550880, Fisher Scientific) to visualize immunoreactivity. Sections were then counterstained in hematoxylin, dehydrated, and cover-slipped. For NF and MBP, fluorescent secondary antibodies (#PIA32731 for Alexa Fluor Plus 488 and #PIA3274 for Alexa Fluor Plus 594, Fisher Scientific) were used at 1:400 dilution. For αSMA staining, incubation of rabbit anti-αSMA antibody was followed by a fluorescent secondary antibody (#PIA3274 for Alexa Fluor Plus 594, Fisher Scientific; 1:400 dilution). To visualize nuclei, 4',6-diamidino-2-phenylindole (DAPI) was used (#62248, Thermo Scientific; 1:1000).

Sections were scanned using a Zeiss Axio Scan Z1 automated slide scanner (Carl Zeiss Spectroscopy GmbH) at 200× magnification for MT and CD68, and 400× for αSMA, NF, and MPB. Slides stained with MT and CD68 were scanned using the bright-field light source. Slides stained with αSMA or double-stained with NF and MBP were scanned using the fluorescent light source. The Zeiss Axio Scan Z1 automated slide scanner rendered a single image for each histology section. Images were collected for the entire sample cross-section. Histologic quantification was performed using ImageJ (National Institutes of Health).

The collagen-to-cell ratio was quantified by adjusting each image's hue, saturation, and brightness levels to capture the relevant blue area that corresponded to the collagen. Using the same function, the manual color threshold was adjusted to select the pink areas corresponding to cellular cytoplasm. An average collagen-to-cell ratio was obtained by dividing the collagen pixel area by the cellular cytoplasm area. To quantify the foamy phagocytes, a manual color threshold was applied to highlight the white areas in the MT staining representing the foamy phagocytic cells. Selected areas were counted using a particle count function, with settings adjusted to include particles of sizes ranging from 5 to 1000 μm and circularity between 0.09 and 1. This particle size adjustment was chosen based on optimization to capture the area of interest. Data were output using ImageJ into an excel sheet for further analysis. The sum of all areas per image was divided by the nerve fascicle area to normalize the data, presented as percent area.

Immunohistochemistry slides were used to quantify CD68+ macrophages, blood vessels, and axons. Quantification of CD68+ macrophages was measured by area, where images were manually thresholded for brown areas corresponding to positive cellular staining. The area of pixels was measured within the cross-sectional areas of the nerves and recorded as the percent of CD68+ macrophages in the total area of nerve fascicles. Immunofluorescence staining for αSMA was employed to identify vascular components within the nerve. These were enumerated within the nerve's cross-sectional areas using the Cell Counter function of ImageJ, and the results were noted as the number of vessels within the nerve fascicle. Axon quantification was achieved by adjusting the manual color threshold to highlight red areas, representing axons, and employing a particle count with settings adjusted to include particles 0–10 μm in size with circularity between 0.2 and 1. ImageJ then generated an excel sheet for each analyzed image, cataloging the number of particles corresponding to the number of axons within the nerve fascicles.

Data were assessed for normal distribution using the D’Agostino and Pearson test before performing statistical tests for differences between groups. A *t* test was used for data with a normal distribution, including gastrocnemius muscle weight ratio, collagen-to-cell ratio, blood vessel counts, and axon counts. A Mann–Whitney U test was used for data with non-normal distribution, including adhesion score, foamy phagocytes, and CD68-stained macrophages. Groups were considered significantly different when the *P* value was less than or equal to .05. Results are reported as mean ± standard deviation (SD) and 95% confidence interval (95% CI).

## Results

**Figure 3:**
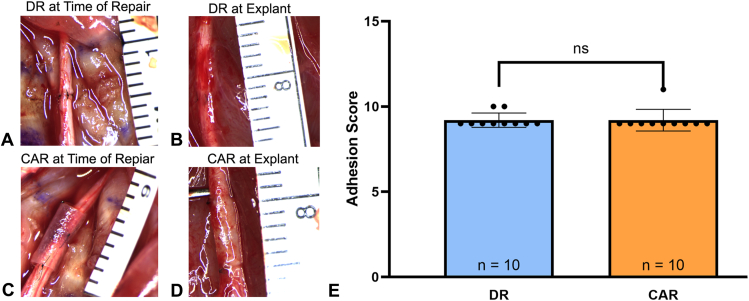
Representative images of the sciatic nerve. **A** Immediately after surgical repair and before closing the surgical site for the DR at time of repair. **B** Six weeks after surgery of the DR at explant. **C** Representative image of the sciatic nerve immediately after surgical repair and before closing the surgical site for the CAR at time of repair. **D** Representative image 6 weeks after surgery of the CAR at explant. **E** Adhesion scores of the DR and CAR groups showing no significant differences between adhesion scores, *P* > .99. Data presented as mean ± SD.

All results were collected 6 weeks after surgery. No adverse reactions were noted throughout the course of the study. There was connective tissue noted at the time of explant surrounding the nerve of all samples, which was not discernible in the explant photos (Fig. 3A–D). Adhesion scores were similar between groups, with the average adhesion scores for the DR group at 9.20 ± 0.42 (95% CI, 8.90–9.50) and for the CAR group at 9.20 ± 0.63 (95% CI, 8.75–9.65, Fig. 3E).

**Figure 4:**
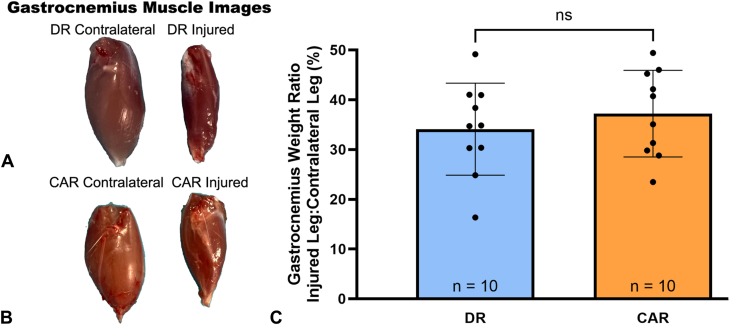
There was visible muscle atrophy in the gastrocnemius muscle explants in both groups. **A** The DR group. **B** The CAR group. **C** The ratio of muscle wet weight of the gastrocnemius of the injured leg to the gastrocnemius of the contralateral leg showed no significant differences between DR and CAR groups, *P* = .45. Data presented as mean ± SD.

Muscle atrophy of the injured leg was visible at explant and similar between groups, with an approximately 65% loss of muscle weight compared with the contralateral, uninjured leg (Fig. 4A–C). Although not notably different, the average ratio of injured leg gastrocnemius to uninjured leg gastrocnemius was lowest in the DR group at 34.10% ± 9.25% (95% CI, 27.48% to 40.72%) and higher in the CAR group was 37.22% ± 8.71% (95% CI, 30.99% to 43.44%).

### Histology

**Figure 5:**
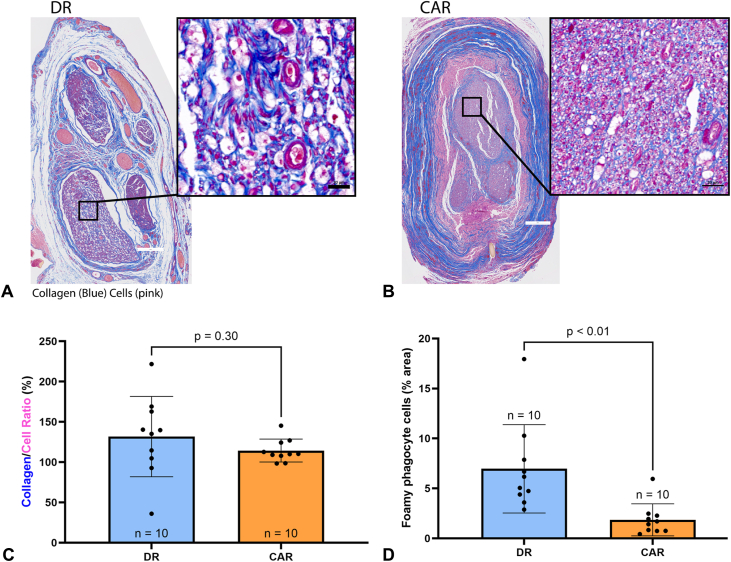
Representative images of the sciatic nerve 6 weeks postsurgery stained with MT. **A** There were notable foamy phagocytes identified in the DR group, characterized by their foamy appearance. The scale bars are at 200 μm in the entire fascicle image (left) and 20 μm in the higher magnification image (right). **B** Foamy phagocytes were less evident in the CAR group. The scale bars are at 200 μm in the entire fascicle image (left) and 20 μm in the higher magnification image (right). **C** Quantitative assessment of collagen-to-cell area ratio. **D** Quantitative assessment of foamy phagocyte cells. Data presented as mean ± SD.

The average collagen-to-cell ratio within the fascicular area was similar between groups. The average collagen-to-cell ratio of the DR group was 131.80% ± 49.80% (95% CI, 96.13% to 167.40%) and 114.40% ± 14.19% (95% CI, 104.30% to 124.60%) for the CAR group (Fig. 5A–C). Cells consistent with foamy phagocytes were observed in the DR group. These foamy phagocytes were identified by a foamy appearance of the cells, suggesting that macrophages have engulfed lipids. The presence of foamy phagocytes is an indicator of on-going Wallerian degeneration.^13^, ^14^, ^15^ After sciatic nerve injury, total macrophage counts peak 14 days after injury and decrease to lower concentrations 28 days after injury.^16^ These macrophages also showed foamy phagocyte characteristics within 2 days after injury, as macrophages were noted to engulf myelin as part of the Wallerian degeneration process.^16^ Quantitative analysis confirmed a considerably larger area of foamy phagocyte cells in the DR group compared to the CAR group, with an average area of foamy phagocyte cells for the DR group of 6.97% ± 4.43% (95% CI, 3.80% to 10.13%) and 1.86% ± 1.60% (95% CI, 0.71% to 3.00%) for the CAR group (*P* < .01, Fig. 5D). This persistent presence of foamy phagocytes is indicative of on-going Wallerian degeneration in the DR group.

**Figure 6:**
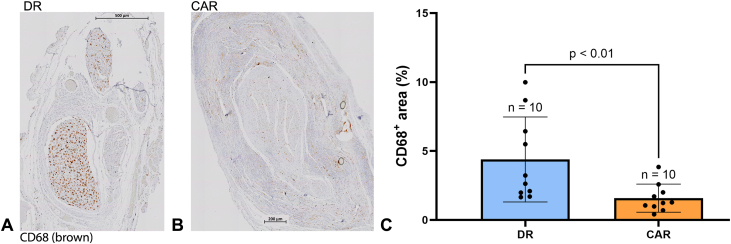
Representative images of the sciatic nerve 6 weeks postsurgery stained with CD68. **A** The DR group (500 μm scale bar). **B** The CAR group (200 μm scale bar). Staining of CD68+ macrophages was more prominent in the DR group than the CAR group. **C** Quantitatively, the DR group had a significantly higher presence of CD68-stained macrophages than that of the CAR group, *P* < .01. Data presented as mean ± SD.

**Figure 7:**
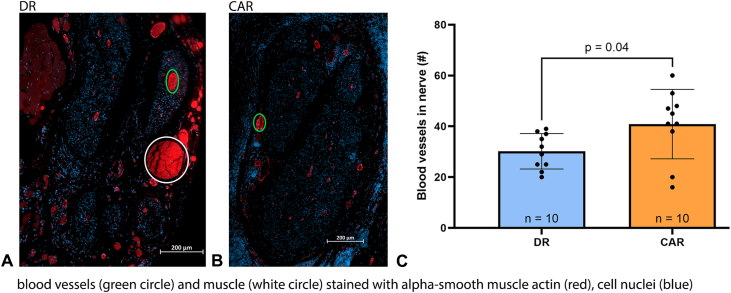
Representative images of immunohistochemical double-staining using αSMA (blood vessels and muscle, red) and DAPI (cell nuclei, blue). **A** The DR group (200 μm scale bar). **B** The CAR group (200 μm scale bar). **C** The number of blood vessels in the CAR group were significantly higher than that of the DR group, *P* = .04. Data presented as mean ± SD.

**Figure 8:**
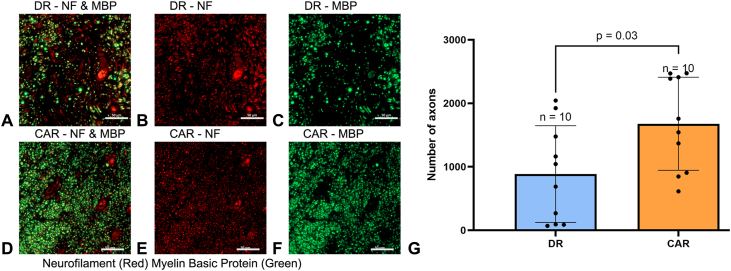
Representative immunohistochemical double-staining images of anti-neurofilament (red) and anti-MBP (green). **A–C** The DR group. **D–F** The CAR groups. The scale bars are at 50 μm. **G** The quantitative assessment showed that the CAR group had a significantly higher axonal count than the DR group, *P* = .03. Data presented as mean ± SD.

Inflammatory cells in this study were quantified within the nerve by staining macrophages with anti-CD68. There was notably higher CD68-staining in the DR group and minimal CD68-staining in the CAR group (Fig. 6A and B). Quantitative analysis showed a considerably larger area of CD68-stained macrophages in the DR group, with the average CD68-stained percent area of the DR group at 4.39% ± 3.08% (95% CI, 2.19% to 6.59%) and 1.58% ± 1.02% (95% CI, 0.86% to 2.31%) for the CAR group (*P* < .01, Fig. 6C). Within the fascicles, there were on average 30.20 ± 6.97 (95% CI, 25.21–35.19) blood vessels in the DR group and 40.90 ± 13.67 (95% CI, 31.12–50.68) blood vessels in the CAR group (Fig. 7A–C). The CAR group had significantly more blood vessels than the DR group (*P* = .04). The NF staining of axons appeared to be more abundant in the CAR group than in the DR group (Fig. 8A–F). Quantitative assessment showed that the CAR group had a considerably higher axonal count than the DR group, with 886 ± 241 (95% CI, 341–1,431) axons in the DR group and 1,678 ± 232 (95% CI, 1,154–2,203) axons in the CAR group (*P* = .03, Fig. 8G). These differences indicated better nerve health in the CAR group than the DR group.

## Discussion

Both injury to the peripheral nerve and its surrounding wound bed may impact peripheral nerve repair outcomes. Although direct nerve injury has the potential to result in intraneural scar formation, injury to the surrounding soft tissue bed may generate extraneural adhesions and negatively affect the inflammatory environment. Isolating the nerve from the surrounding environment through the use of a nerve protection device has been shown to reduce the likelihood of adhesions to the nerve.^17^ The present study investigated the effects of protecting the coaptation site using a nerve conduit as a coaptation aid on muscle atrophy, extraneural tissue adhesions, intraneural collagen-to-cell ratio, macrophage inflammatory response, vascularization, and axon counts in a rat model after nerve transection and surrounding soft tissue injury. Our investigation found no differences between DR and CAR groups with respect to adhesion score, gastrocnemius muscle weight of the injured leg, and intraneural collagen-to-cell ratio. However, considerable differences were noted between groups with respect to foamy phagocytic cells, CD68+ macrophages, vascularization, and number of axons.

Both DR and CAR groups exhibited similar decreased muscle weight compared to the uninjured contralateral leg, which was attributed to the induced nerve injury and the time point chosen for the assessment. Additionally, there were no notable differences in extraneural adhesions between the DR and CAR group. This may be a secondary effect of the limitations of the employed scoring criteria. The adhesion scoring was cumulative for adhesion quality, tenacity, and extent of site involvement. This cumulative scoring system did not allow for individual assessment of extraneural adhesion characteristics. The lack of granularity of this adhesion scoring system is a limitation of this study, as enhanced resolution and visualization of the samples seen in our study may have permitted a more precise and detailed assessment of the perineural environment. Future studies should use an updated scoring system, which may ensure better differentiation between groups. Furthermore, differentiation between groups with respect to adhesion score may also have been achieved if assessments were performed a later timepoint, further out from injury induction and nerve repair. Future studies should alter the adhesion scoring criteria or evaluate the adhesion scoring at later timepoints.

Although gross pathology did not show considerable differences between groups, microscopic evaluation of stained histology sections showed differences between DR and CAR treatment of nerve injuries. Histological characterization revealed that the DR group exhibited a higher density of CD68+ macrophages and a higher density of foamy phagocytes than in the CAR group. Additionally, the concentrations of CD68+ macrophages and foamy phagocytes were similar within groups, suggesting that the CD68+ macrophages consisted of primarily foamy phagocytes. The higher concentration of CD68+ macrophages and foamy phagocytes in the DR group suggests unresolved Wallerian degeneration. The resolution of Wallerian degeneration is critically important in nerve regeneration and is highly dependent on containing proliferating Schwann cells and the neurotropic milieu between the proximal and distal nerve stumps, which can be supported by the use of a nerve connector.^18^

Further support of the use of CAR was observed in the vascularization and axon counts, where the CAR group exhibited substantially more vascularization and higher axon counts compared to that of the DR group. The presence of higher vascularization has been shown to facilitate axonal regeneration, where Schwann cell migration and axon regeneration have been shown to be the highest in well-vascularized tissue beds.^19^ These findings highlight the potential benefits of CAR in protecting the nerve during the healing process including reduced inflammation, better vascularization, and increased axon counts.

Clinically, nerve injury often occurs alongside damage to surrounding tissues, reflecting the complexity of these traumatic injuries. This co-occurrence challenges treatment strategies, as both nerve and adjacent tissue health must be addressed. DR has been the historical standard of nerve repair when a nerve gap may be overcome by neurolysis or re-approximation without undue tension. Our study highlights the potential benefits of CAR over DR alone to protect the nerve coaptation from the surrounding injured tissue during regeneration by improving surrogate markers of nerve health, including considerably higher axon counts. Higher axon counts may have better clinical outcomes.^20^ Although animal studies cannot be directly predictive of clinical efficacy, the results show promise when considering the clinical application of connector-assisted nerve repairs. The clinical benefits of CAR relative to DR were demonstrated in a recent meta-analysis, which showed higher rates of meaningful recovery measured with Medical Research Council Classification scores.^21^ The histological data presented in our study in conjunction with the clinical outcomes in Leis et al,^21^ where there were enhanced rates of meaningful recovery with CAR over DR, provide promising evidence and a strong basis for preferential use of CAR rather than suture-only DR.

## Conflicts of Interest

Dr Zoghoul Alsmadi is an Axogen shareholder and full-time employee. Dr Deister is an Axogen shareholder and full-time employee. Dr Ghanem serves on the advisory board for Axogen. Dr Mercer has a financial association with Axogen. Dr Evans is a consultant for Axogen. Dr Smetana is a consultant for Axogen, is on the advisory board, and has received previous research funding. This study's research was conducted with the assistance of Axogen Corporation. Bias in this study was controlled by statistical evaluation of histology sections prepared from nerve samples.
